# Texture-Modified Diet for Improving the Management of Oropharyngeal Dysphagia in Nursing Home Residents: An Expert Review

**DOI:** 10.1007/s12603-020-1377-5

**Published:** 2020-05-04

**Authors:** María D. Ballesteros-Pomar, A. Cherubini, H. Keller, P. Lam, Y. Rolland, S.F. Simmons

**Affiliations:** 1 Department of Endocrinology and Nutrition, Complejo Asistencial Universitario de León, León, Spain; 2 Geriatria, Accettazione geriatrica e Centro di ricerca per l'invecchiamento, IRCCS INRCA, Ancona, Italy; 3 Schlegel-University of Waterloo Research Institute for Aging and Department of Kinesiology, University of Waterloo, Waterloo, Canada; 4 Faculty of Land and Food Systems, University of British Columbia, Vancouver, Canada; 5 Department of Geriatrics, Gerontopole de Toulouse, CHU de Toulouse, INSERM 1027, Toulouse, France; 6 Division of Geriatrics, Center for Quality Aging, Vanderbilt University Medical Center, Nashville, Tennessee, USA

**Keywords:** Dysphagia, malnutrition, nursing home, texture-modified diet

## Abstract

**Objectives:**

This paper provides evidence-based and, when appropriate, expert reviewed recommendations for long-stay residents who are prescribed texture-modified diets (TMDs), with the consideration that these residents are at high risk of worsening oropharyngeal dysphagia (OD), malnutrition, dehydration, aspiration pneumonia, and OD-associated mortality, poorer quality of life and high costs.

**Design:**

Nestlé Health Science funded an initial virtual meeting attended by all authors, in which the unmet needs and subsequent recommendations for OD management were discussed. The opinions, results, and recommendations detailed in this paper are those of the authors, and are independent of funding sources.

**Setting:**

OD is common in nursing home (NH) residents, and is defined as the inability to initiate and perform safe swallowing. The long-stay NH resident population has specific characteristics marked by a shorter life expectancy relative to community-dwelling older adults, high prevalence of multimorbidity with a high rate of complications, dementia, frailty, disability, and often polypharmacy. As a result, OD is associated with malnutrition, dehydration, aspiration pneumonia, functional decline, and death. Complications of OD can potentially be prevented with the use of TMDs.

**Results:**

This report presents expert opinion and evidence-informed recommendations for best practice on the nutritional management of OD. It aims to highlight the practice gaps between the evidence-based management of OD and real-world patterns, including inadequate dietary provision and insufficient staff training. In addition, the unmet need for OD screening and improvements in therapeutic diets are explored and discussed.

**Conclusion:**

There is currently limited empirical evidence to guide practice in OD management. Given the complex and heterogeneous population of long-stay NH residents, some ‘best practice' approaches and interventions require extensive efficacy testing before further changes in policy can be implemented.

## Introduction

### The context of the NH

The long-stay NH resident population has specific characteristics marked by a shorter life expectancy relative to community-dwelling older adults, high prevalence of multimorbidity with a high rate of complications, dementia, frailty, disability, and often polypharmacy. Oropharyngeal dysphagia (OD), defined as the inability to initiate and perform safe swallowing (1, 2), alongside poor coordination and propulsion, may result in aspiration from the passing of food, a liquid bolus, or regurgitated gastric contents into the respiratory tract, and is believed to be common among this population (3). This population also has a significantly greater risk of malnutrition, resulting from a variety of challenges including the oral processing of food (4).

Due to the large heterogeneity within the NH resident population, defined resident-specific care needs, including nutritional goals, are urgently required. Personalized resident-centered care planning needs to focus on the management or care of OD.

The aim of this paper is to present an expert-reviewed report on evidence-based optimal practice and positions on the nutritional management of OD where this evidence is available, and a collective opinion on better practices to manage OD in NH residents where empirical evidence is absent. This paper has a specific focus on the role of texture modified diets (TMDs), and on unmet needs that should be addressed in several areas of care and research concerning OD. TMD will be the term used to describe the therapeutic diet prescription used to manage OD.

### Malnutrition and OD in complex residents

Currently, there is a high prevalence of malnutrition in the NH population, with levels ranging from 1.5% to 66.5% depending on how malnutrition is diagnosed (4); OD is associated with a higher risk of malnutrition, which further exacerbates OD (5). Both OD and malnutrition are complex conditions that can result from factors presented in Table 1. Expert recommendations to overcome these unmet needs are presented below.

**Table 1:** Challenges meeting the needs of NH* residents with OD^†^

**Area**	**Unmet need**
Staffing	Inadequate NH staffing(6)
	Lack of nutrition and OD specialists^a^
Diet	Inadequate nutritional intake of some macro and micronutrients in TMD (7, 8)
	Inconsistent daily care provision for eating and drinking assistance (7)
	Resident preferences that may be divergent from dietary needs related to one or more chronic conditions (9, 10)
	NH resident eating challenges (9)
	Inadequate equipment to support interventions^a^
Care quality	Insufficient knowledge of the NH staff, and/or training of care providers within the NH care environment to support the accurate assessment of OD and malnutrition
	Difficulty meeting resident dietary preferences and eating needs in a communal dining environment
	Changing resident needs for eating assistance^a^


*NH, nursing home; ^†^OD, oropharyngeal dysphagia; ^a^Authors' personal opinion based on expert-reviewed recommendation

### Nutritional care: Expert-reviewed recommendations and summary of prior guidelines

Based on the authors' personal opinion, optimal nutritional care of the NH population should be considered a high priority, similarly to wound care. Table 2 provides an overview of optimal practice. The nutritional content of diets, amount, and quality of assistance to eat and drink, the dining environment, and the quality of oral hygiene are important factors to consider for analyzing the NH population.

**Table 2:** Recommendations to achieve optimal nutritional care in OD* management

Potential ways to improve nutritional care in nursing home residents
Perform screening and periodic reassessments of both dysphagia and the risk of malnutrition, and dehydration (23, 24)
Plan and conduct multimodal interventions and rehabilitation (25, 25)
Support the communication between the multidisciplinary team and dysphagia clinician regarding the recommendations and nutritional treatment plans (17)
Involve a dietitian and food service manager to plan and provide a diet that meets nutritional needs.
Assist residents with exercise (e.g. mobility interventions); this is frequently recommended with nutritional interventions with the aim to improve strength and decrease frailty (26, 27)
Provide specialized nutrition support as required (28, 29)
Perform a regular medication review, with the aim of reducing medications that can negatively impact appetite or alertness. Also perform a periodic training review for emergency situations, such as a case of aspiration and acute dyspnea due to OD
Ensure that there is at least one licensed supervisor present during mealtimes to oversee mealtime care quality, monitor symptoms of OD, and respond to acute needs.


*OD, oropharyngeal dysphagia

Although routine oral hygiene and dental care are necessary to reduce the risk of aspiration pneumonia infections, and improve chewing and oral sensory processing, these aspects of personal care are often not managed adequately (11) (Authors' personal opinion based on expert-reviewed recommendation). It is also important that NH residents who are able to, should leave their bed for most of their meals as this reduces the risk of poor oral intake and swallowing difficulties from poor positioning (12).

Residents receiving medication (either single drugs or polypharmacy) should be carefully monitored as these residents may have reduced appetites that may contribute to malnutrition-associated complications (13, 14) (Authors' personal opinion based on expert-reviewed recommendation). A regular medication review should be performed for these residents to carefully evaluate the necessity of each medication, particularly those that can suppress appetite, lead to mouth dryness, and/or have sedative side effects. Resident-specific goals should be developed with the aim of considering the individual's wishes and the trajectory of their health status, rather than treating the symptoms alone.

The goals for most residents should be to meet fluid, macronutrient, and micronutrient recommendations to maintain the best possible health status and contribute to the prevention of falls, fractures, functional decline, pressure ulcers, and infections. Evidence from previously published guidelines and expert-reviewed recommendations for the nutritional care of NH residents are summarized in Supplementary Tables 1 and 2. However, it should be acknowledged that quality of life must be the priority goal in residents with a short life expectancy, and/or those who otherwise express a preference for quality of life rather than prolonged life. For these residents in particular, management goals should optimize satisfaction from food and their mealtime experience. Consequently, the necessity of dietary restrictions, such as salt and concentrated sugar exclusions, and/or the reduction of fat, should be carefully evaluated among these residents.

As long-stay NH residents tend to suffer from various health conditions that can lead to or exacerbate OD, it is also important to consider the nutritional requirements specific to health conditions that are often present in those with OD (Supplementary Tables 1 and 2). NH residents often suffer from more than one condition, thus, personalized recommendations should be provided that consider residents' specific needs; a comprehensive nutrition assessment is usually applied to determine the optimal treatment/management plan for a given resident.

### Gaps in practice between evidence-based management of OD and real-world patterns

The recommended therapeutic approaches for the management of OD include swallowing therapy and training, TMDs, and technical methods, such as peripheral stimulation and transcranial direct-current stimulation (15). Although TMDs are recommended as a management strategy for OD, evidence supporting their benefits on clinical outcomes is lacking due to few studies and small sample sizes (15, 16, 17).

#### Inadequate dietary provision: Menu planning and recipe development

The current management strategy of NH residents often differs to various guideline recommendations (Supplementary Tables 1 and 2). Although recommended to manage OD, TMDs provided for NH residents when inappropriately prepared, may be lower in key nutrients than regular diets (16, 18). Adequate amounts of protein, fiber, liquid, and key nutrients such as vitamins D, K and E, folate, magnesium, calcium, potassium, and zinc are the most difficult to achieve (19, 20). A nutrient-poor diet poses a risk factor for infections, and insufficient liquid intake may lead to dehydration, which may in turn affect the degree of responsiveness and functioning of the oropharyngeal tract (21). However, research has shown that these issues can be mitigated to some extent by good-quality menus and recipes (18), and hydration protocols (22), yet high-quality intervention studies are lacking.

### Insufficient NH staff and staff training

The following recommendations are based on the authors' opinions. A lack of resources often translates into insufficient levels of staffing in the NH setting and the inability to form a multidisciplinary team to prepare and perform appropriate nutritional care plans, and monitor nutrition and hydration statuses. Currently, a knowledge gap often exists among NH staff regarding residents' nutrition and hydration needs, the screening and monitoring of OD, malnutrition, the preparation and use of TMDs, and how to address eating challenges, which complicate unified training approaches. Supplementary Table 3 lists the potentially unmet needs identified by the committee that influence the diagnosis and management of OD. NH staff, professional caregivers, and home helpers should be provided with simple training in the management and detection of OD, malnutrition, and dehydration. Supplementary Table 4 provides recommendations for staff training.

**Table 3:** Recommendations for the improvement of TMDs^†^

Residents recommended by their dysphagia clinician to follow a TMD should be referred to a nutritional professional (23, 24).
TMD/thickened liquids and nutrient-dense, or fortified foods are ‘therapeutic diets' (25). Specially-made, nutritionally-enriched texture-modified foods, and thickened liquids should be recommended for older adults with chronic dysphagia for improving nutritional status, promoting hydration, and minimizing the risk of aspiration (17, 31)^a^.
Foods should be enhanced with energy- and protein-dense ingredients to prevent declines in nutritional and functional status (26, 32, 33). Nutritional analysis of TMDs ensures the comparability of products with regular offerings. Consider ingredient adjustments to increase micronutrient levels (34).
When food intake is inadequate, consider ONS* to improve energy and protein intake, preferably offered as 50–125 mL with medication rounds, multiple times/day, between meals and with the encouragement of carers (requires 5–10 minutes/person/offer) (26, 35). ONS provides 400+ kcal/day, including 30+ g protein/day for those at risk or malnourished (36).
Adjust portion sizes as per the individual's needs and preferences. Offer a variety of options (flavors, optimal texture and thickness ranges based on the individual's abilities) to the greatest extent possible to decrease rate of refusal.
To reduce mealtime fatigue and its associated risk of aspiration, volume of sip or spoon size should be adjusted as per personal requirements.
Aim to provide adequate food/fluid according to individual needs and preferences, considering their wishes and safety (28).
Increase food attractiveness(15) by presenting in a visually-appealing and dignified way (31, 32, 37). Texture-modified food does not necessarily mimic the appearance of unmodified food, as evidence is lacking for molded foods (38)^a^.
Increase diversity and offer a range of alternative meal choices, and protein- and energy-dense in-between meal snacks to provide adequate nutrition, improve resident autonomy and engagement, and encourage consumption (39).
Personalize the ambiance and delivery method to promote appetite and food intake (26, 27, 40).
Ensure the use of TMDs and the intake of texture-modified foods, and the resident's nutritional status, are closely monitored and evaluated.
Encourage eating but do not force food on residents, as this may take away pleasure from a person's mealtime and lead to reduced food intake, and/or quality of life. Proactively address eating challenges and provide tailored support (e.g. more time to eat, quiet environment, finger foods, etc.).
Apply the International Dysphagia Diet Standardization Initiative framework in the prescription, preparation, and assessment of TMDs and thickened liquids to facilitate consistent communication between nursing home staff, carers, and professionals, and reduce variability in the preparation, assessment, and prescription of TMDs and thickened liquids (41).
Rapidly and thoroughly evaluate the resident when he/she presents anorexia or refusal to eat while treated with a TMD; the use of TMDs should be re-evaluated in the context of end-of-life and palliative care.


*ONS, oral nutritional supplements; ^†^TMD, texture-modified diet. ^a^Authors' personal opinion based on expert-reviewed recommendation

### Unmet needs: Screening and management

To address the unmet needs in OD screening and management, it is recommended that a multidisciplinary team of professionals is involved, who can ensure optimal nutritional care that prevents aspiration, malnutrition, and dehydration. There are a number of ways in which this can be done, as listed (Table 2).

### Unmet need: Improvements to the quality of therapeutic diets

In the absence of strong evidence for TMDs to promote safety and beneficial long-term outcomes (30), it is the collective opinion of the committee that TMDs when managed and developed appropriately, and accepted by the consumer, can benefit the resident. It is also important to note the lack of research and where present, inconsistencies between studies that call for improvements in TMD standardization (30). Based on the opinion of the authors, adherence to nutritionally-enhanced TMDs may have a direct effect on unintentional weight loss, dehydration, skin health, wound healing, and quality of life of NH residents, and may additionally exhibit a positive impact on functional outcomes, such as improved mobility and independence when TMDs are used, combined with other interventions (e.g. mobility interventions or other forms of exercise). Recommendations for the improvement and consistency of nutritional quality, and improved adherence to TMDs for residents with swallowing difficulties are listed in Table 3. Rigorous intervention studies examining the efficacy of the various strategies are required to provide evidence to support these recommendations.

### Unmet need: Minimal training requirements

To ensure that NH residents are given the required nutritional support, the minimum training requirements outlined (Supplementary Table 4) are recommended by the committee for NH staff.

Budget resource constraints may account for a large part of the training and staffing resource deficiencies that exist in many NHs. In order to alleviate budget constraints, solutions such as: Cross-training of additional personnel to assist with nutritional care delivery in the NH; sharing of resources (both equipment and specialized personnel) across different NHs, and between NHs and hospitals; and virtual assessment tools may be employed as cost-effective strategies to improve nutrition and OD care quality with existing resources (42). As the rate of death in NHs is high, all staff should be integrated into the process of making recommendations for palliative care for residents. As an optimal measure, staff should also consider patient wishes, and give priority to the quality of life for residents.

## Summary

The population of long-stay NH residents is heterogeneous and complex, and is often characterized by the presence of one or more chronic conditions with the addition of a high risk of OD, malnutrition, and/or dehydration. Due to insufficient resources, inadequate staff training and a lack of collective opinion on best practices, there is often a suboptimal approach to nutrition, hydration and OD care in NHs (4). This paper presents, where available, evidence-based recommendations aimed to support NH staff and multidisciplinary teams in the preparation and execution of personalized nutritional care plans for long-stay NH residents prescribed TMDs to manage OD. A list of evidence-informed and expert recommendations on nutritional requirements, improvements to TMDs and their provision, and a checklist of minimal training requirements for NH staff have been provided. However, it should be noted that the research is lacking in this area and a systematic review of the literature was not possible. Some recommendations provided that have been considered as ‘best practices' are yet to be demonstrated as effective for improving the health and quality of life for NH residents. The committee also recommends a concerted effort in the development and testing of efficacious interventions to develop the evidence base for this population.

### Implications for practice, policy, and/or research

This report presents expert opinion and evidence-informed recommendations for optimal practice and positions on the nutritional management of OD. In addition, this report provides expert guidance for improved OD management in NH residents. Given the complex and heterogeneous population of long-stay NH residents, some ‘best practice' approaches and interventions will require rigorous efficacy testing before further changes in policy can be implemented.
