# Supplementary material for: Texture-Modified Diet for Improving the Management of Oropharyngeal Dysphagia in Nursing Home Residents: An Expert Review
Source: J Nutr Health Aging. 2020 May 4;24(6):576–81. doi: 10.1007/s12603-020-1377-5 (PMC12876721; doi:10.1007/s12603-020-1377-5)
Supplement: Supplementary file 1 — Supplementary Material [file mmc1.docx]

## **Supplementary Material**

## **Table 1: OD**^‡‡^ **Related condition-specific evidence and expert recommendations for nursing home residents**

| Condition | Recommendation |
| --- | --- |
| ALS^*^, PD^\|\|^, MS^‡^, stroke, dementia | Generally: Nutritional status assessment and screening for dysphagia(1) as many neurological conditions will affect oral processing and swallowing capacity, and malnutrition is common. TMDs^††^ and use of ONS^§^ are common strategies.(2)  Other specific recommendations:  ALS: Approximately 30 kcal/kg BW^†^/day (depending on physical activity); ONS if required(1)  PD: Careful monitoring of BW, folic acid, and vitamins D and B_12_(1)  MS: Diet low in saturated fats(1)  Stroke: Consider TMD and thickened fluids if OD is present(1)  Dementia: Recommendations should be made on an individual basis taking into account potential health benefits and impacts on quality of life, (3) as well as long term use of TMD(4) |
| Sarcopenia | Protein: 1.0–1.5 g/kg BW/day,(5) leucine-enriched balanced essential amino acid mix may be added (6)  Monitor vitamin D supplementation, particularly in those with low baseline levels(7)  Obese residents should follow a balanced diet |
| Frailty | Protein: 1.2–1.5 g/kg BW/day(8)  Calorie intake, protein quality, and vitamin D status to be checked by clinicians; ONS may be considered(9) |
| Fall / fracture risk | Balanced diet (including calcium intake)(10)  Vitamin D: Maintain serum levels >50 nmol/L (associated with a 20% reduction in falls and fractures)(11) |
| Malnutrition | Protein: 1.2–1.5 g/kg BW/day(11)  Energy: >1500 kcal/day(11)  Vitamin D: Maintain a minimum serum level of 65 nmol/L(11)  Obese residents should follow a balanced diet |
| Dehydration | Fluid: >1700 mL/day(11)^–^(12)  1. 6L Females and 2.1 L males unless clinical condition indicates otherwise(13) |
| Kidney insufficiency | PROT-AGE^**^ recommends a protein intake of 1.0–1.2 g/kg BW/day, citing no evidence of effect on renal function(8) |
| Pressure ulcers | Higher fluid and protein intake – via protein supplementation with a minimum of 30 g/d of protein - has been shown to significantly improve the healing of pressure ulcers(14)^a^ |
| Obesity | Intentional weight loss is not a goal in obese residents if they are at risk of either malnutrition or sarcopenia; instead, a balanced diet is recommended |

^*^ALS, amyotrophic lateral sclerosis; ^†^BW, body weight; ^‡^MS, multiple sclerosis; ^§^ONS, oral nutritional supplements;
^||^PD, Parkinson’s disease; ^**^PROT-AGE, protein needs with ageing; ^††^TMD, texture-modified diet; ^‡^OD, oropharyngeal dysphagia. ^a^Authors’ personal opinion, based on expert-reviewed recommendation

## **Table 2: General nutritional recommendations for the older population and nursing home residents**

| Nutrient | Recommendation |
| --- | --- |
| Energy | SENC^††^ recommends an average of 30 kcal/kg BW^*^/day,(15) ESPEN^‡^ recommends 27 kcal/kg BW/day for individuals with polymorbidity(16) |
| Protein | PROT-AGE^**^ Study Group recommends an average daily intake of 1.0–1.2 g protein/kg BW/day, with minimum 25–30 g protein per meal;(8) up to 1.5 g protein/kg BW/day(17) |
| Fibre | SENC recommends 25–30 g/day,(15)^,^(18) WHO recommends >25 g/day(19) |
| Fluid | EFSA^†^ recommends 2000–2500 mL/day (~80% from beverages)(20) |
| Calcium | 1200–1500 mg/day (in addition to vitamin D)(21; 22) |
| Vitamin D | IOF^§^ recommends 20–25 μg/day (800–1000 IU) to reach a target serum 25-hydroxyvitamin D level of 75 nmol/L(21; 23) |
| Vitamin B_12_ | IOM^\|\|^ recommends a dietary allowance of 2.4 µg/day of vitamin B_12_ for adults aged >50 years, and advises that most of this nutrient can be obtained by consuming foods fortified with B_12_, or a B_12_-containing supplement(24) |
| Food texture/Liquid thickness | Confirm the food texture/liquid thickness modifications based on clinical swallowing assessment. Food and liquids are adapted to facilitate swallowing safety and maximize food intake. The International Dysphagia Diet Standardisation Initiative recommends eight levels (0-7), where drinks are measured from levels 0–4, while foods are measured from levels 3–7(25) |

^*^BW, bodyweight; ^†^EFSA, European Food Safety Authority; ^‡^ESPEN, European Society for Clinical Nutrition and Metabolism;
^§^IOF, International Osteoporosis Foundation; ^||^IOM, Institute of Medicine; ^**^PROT-AGE, protein needs with ageing;
^††^SENC, Spanish Society of Community Nutrition); WHO, World Health Organization

## **Table 3: Committee’s views on unmet needs for OD^†^ diagnosis and management**

| Area | Unmet need |  |
| --- | --- | --- |
| Perceptions and attitudes | Cultural perception of OD and older adult care may lead to inaction. | |
|  | NH^*^ staff and healthcare practitioners may feel pressured to ensure the achievement of nutrient dietary reference intake targets, without acknowledging the resident’s quality of life | |
|  | Dysphagia can be an end-of-life symptom; however, this does not negate the need for assessment and the potential appropriateness of intervention | |
| Diagnosis | Inadequacy in the level and frequency of assessment impedes the prevention of OD-associated complications | |
|  | Due to the lack of consistent screening and standards of care, it is unclear which professional should be in charge, and which tools should be used in the different stages of the screening/diagnosis process | |
|  | Lack of staffing and/or instrumental resources required for appropriate and timely diagnostic assessment of OD is an ongoing problem | |
| Management | One-size-fits-all approach cannot meet the needs resulting from the complex and diverse manifestations of OD | |
|  | Lack of staffing resources and unavailability of an appropriate multidisciplinary team of experts impede the assurance of:   - Proactive action to prevent malnutrition, dehydration and unintentional weight loss in NH residents with OD - Adequate eating assistance; it is suggested that 35–40 min/person/meal is needed for safe eating assistance(26) | |

## ^*^NH, nursing home; ^†^OD, oropharyngeal dysphagia

**Table 4: Recommendations for the minimum training requirements for NH^*^ staff**

| Conceptual/knowledge-based |
| --- |
| Basic physiology of swallowing and changes in swallowing associated with ageing |
| Difficulties posed by OD^†^ and malnutrition in NH residents |
| Concepts, strategies of nutrient-rich foods to meet nutrient dietary reference intake |
| Concepts and strategies of nutrition in the context of end-of-life and palliative care |
| Concepts, standards, and strategies of food texture and fluid thickness modification |
| Polypharmacy, side effects of drugs (e.g. on swallowing difficulty, appetite, alertness) |
| **Practical** |
| Screening, evaluating and monitoring OD:(27)   - To detect those at risk through a validated dysphagia screening tool - Clinical assessment to evaluate the efficacy and safety of swallowing - Instrumental assessment (where available) to confirm diagnosis - All assessments should be standardized among clinicians to avoid subjective  clinician judgement |
| Screening/evaluating and monitoring malnutrition:(28)   - Screening to detect those at risk using tools appropriate for NH populations - Nutritional assessment comprising medical history, weight history, food intake, function, physical examinations, biochemical evaluation to diagnose malnutrition |
| Monitoring daily food and fluid intake using standardized mealtime observation and estimation methods, or standardized weighing protocols |
| Providing mealtime management support which may include physical and verbal eating assistance cues; how to support self-feeding for as long as possible in a flexible manner that is dignified(26; 29) |
| Providing sensory appeal and pleasure through food and therapeutic diets |
| Performing International Dysphagia Diet Standardisation Initiative fork pressure, fork drip, and spoon tilt tests to evaluate TMDs(30)^‡^ |
| Comprehensive assessment and management of complex needs |
| Roll out methodology of multidisciplinary support to proactively evaluate, and monitor OD and food intake |
| Support and assist with regular oral and dental care |

^*^NH, nursing home; ^†^OD, oropharyngeal dysphagia; ^‡^TMD, texture-modified diet

**Supplementary References**

1. Burgos R, Breton I, Cereda E, Desport JC, Dziewas R, Genton L, Gomes F, Jesus P, Leischker A, Muscaritoli M, Poulia KA, Preiser JC, Van der Marck M, Wirth R, Singer P, Bischoff SC (2018) ESPEN guideline clinical nutrition in neurology. Clin Nutr 37 (1):354-396. doi:10.1016/j.clnu.2017.09.003
2. Sura L, Madhavan A, Carnaby G, Crary MA (2012) Dysphagia in the elderly: management and nutritional considerations. Clin Interv Aging 7:287-298. doi:10.2147/CIA.S23404
3. Volkert D, Chourdakis M, Faxen-Irving G, Fruhwald T, Landi F, Suominen MH, Vandewoude M, Wirth R, Schneider SM (2015) ESPEN guidelines on nutrition in dementia. Clin Nutr 34 (6):1052-1073. doi:10.1016/j.clnu.2015.09.004
4. Flynn E, Smith CH, Walsh CD, Walshe M (2018) Modifying the consistency of food and fluids for swallowing difficulties in dementia. Cochrane Database Syst Rev 9:CD011077. doi:10.1002/14651858.CD011077.pub2
5. Yoshimura Y, Bise T, Shimazu S, Tanoue M, Tomioka Y, Araki M, Nishino T, Kuzuhara A, Takatsuki F (2019) Effects of a leucine-enriched amino acid supplement on muscle mass, muscle strength, and physical function in post-stroke patients with sarcopenia: A randomized controlled trial. Nutrition 58:1-6. doi:10.1016/j.nut.2018.05.028
6. Morley JE, Argiles JM, Evans WJ, Bhasin S, Cella D, Deutz NE, Doehner W, Fearon KC, Ferrucci L, Hellerstein MK, Kalantar-Zadeh K, Lochs H, MacDonald N, Mulligan K, Muscaritoli M, Ponikowski P, Posthauer ME, Rossi Fanelli F, Schambelan M, Schols AM, Schuster MW, Anker SD, Society for Sarcopenia C, Wasting D (2010) Nutritional recommendations for the management of sarcopenia. J Am Med Dir Assoc 11 (6):391-396. doi:10.1016/j.jamda.2010.04.014; Deutz NE, Bauer JM, Barazzoni R, Biolo G, Boirie Y, Bosy-Westphal A, Cederholm T, Cruz-Jentoft A, Krznaric Z, Nair KS, Singer P, Teta D, Tipton K, Calder PC (2014) Protein intake and exercise for optimal muscle function with aging: recommendations from the ESPEN Expert Group. Clin Nutr 33 (6):929-936. doi:10.1016/j.clnu.2014.04.007
7. De Spiegeleer A, Beckwee D, Bautmans I, Petrovic M, Sarcopenia Guidelines Development group of the Belgian Society of G, Geriatrics (2018) Pharmacological Interventions to Improve Muscle Mass, Muscle Strength and Physical Performance in Older People: An Umbrella Review of Systematic Reviews and Meta-analyses. Drugs Aging 35 (8):719-734. doi:10.1007/s40266-018-0566-y
8. Bauer J, Biolo G, Cederholm T, Cesari M, Cruz-Jentoft AJ, Morley JE, Phillips S, Sieber C, Stehle P, Teta D, Visvanathan R, Volpi E, Boirie Y (2013) Evidence-based recommendations for optimal dietary protein intake in older people: a position paper from the PROT-AGE Study Group. J Am Med Dir Assoc 14 (8):542-559. doi:10.1016/j.jamda.2013.05.021
9. Beaudart C, McCloskey E, Bruyere O, Cesari M, Rolland Y, Rizzoli R, Araujo de Carvalho I, Amuthavalli Thiyagarajan J, Bautmans I, Bertiere MC, Brandi ML, Al-Daghri NM, Burlet N, Cavalier E, Cerreta F, Cherubini A, Fielding R, Gielen E, Landi F, Petermans J, Reginster JY, Visser M, Kanis J, Cooper C (2016) Sarcopenia in daily practice: assessment and management. BMC Geriatr 16 (1):170. doi:10.1186/s12877-016-0349-4
10. Blain H, Masud T, Dargent-Molina P, Martin FC, Rosendahl E, van der Velde N, Bousquet J, Benetos A, Cooper C, Kanis JA, Reginster JY, Rizzoli R, Cortet B, Barbagallo M, Dreinhofer KE, Vellas B, Maggi S, Strandberg T, Falls E, Fracture Interest G, European Society for C, Economic Aspects of O, Osteoarthritis OR, Information G, International osteoporosis F (2016) A Comprehensive Fracture Prevention Strategy in Older Adults: The European Union Geriatric Medicine Society (EUGMS) Statement. J Nutr Health Aging 20 (6):647-652. doi:10.1007/s12603-016-0741-y
11. van Asselt DZ, van Bokhorst-de van der Schueren MA, van der Cammen TJ, Disselhorst LG, Janse A, Lonterman-Monasch S, Maas HA, Popescu ME, Scholzel-Dorenbos CJ, Sipers WM, Veldhoven CM, Wijnen HH, Olde Rikkert MG (2012) Assessment and treatment of malnutrition in Dutch geriatric practice: consensus through a modified Delphi study. Age Ageing 41 (3):399-404. doi:10.1093/ageing/afs005
12. Botigue T, Masot O, Miranda J, Nuin C, Viladrosa M, Lavedan A, Zwakhalen S (2019) Prevalence and Risk Factors Associated With Low Fluid Intake in Institutionalized Older Residents. J Am Med Dir Assoc 20 (3):317-322. doi:10.1016/j.jamda.2018.08.011
13. Volkert D, Beck AM, Cederholm T, Cruz-Jentoft A, Goisser S, Hooper L, Kiesswetter E, Maggio M, Raynaud-Simon A, Sieber CC, Sobotka L, van Asselt D, Wirth R, Bischoff SC (2019) ESPEN guideline on clinical nutrition and hydration in geriatrics. Clin Nutr 38 (1):10-47. doi:10.1016/j.clnu.2018.05.024
14. Breslow RA, Hallfrisch J, Guy DG, Crawley B, Goldberg AP (1993) The Importance of Dietary Protein in Healing Pressure Ulcers. Journal of American Getriatrics Society 41:357-362; Namasivayam-MacDonald AM, Slaughter SE, Morrison J, Steele CM, Carrier N, Lengyel C, Keller HH (2018) Inadequate fluid intake in long term care residents: prevalence and determinants. Geriatr Nurs 39 (3):330-335. doi:10.1016/j.gerinurse.2017.11.004; Keller HH, Lengyel C, Carrier N, Slaughter SE, Morrison J, Duncan AM, Steele CM, Duizer L, Brown KS, Chaudhury H, Yoon MN, Boscart V, Heckman G, Villalon L (2018) Prevalence of inadequate micronutrient intakes of Canadian long-term care residents. Br J Nutr 119 (9):1047-1056. doi:10.1017/S0007114518000107
15. Arbones G, Carbajal A, Gonzalvo B, Gonzalez-Gross M, Joyanes M, Marques-Lopez I, Martin ML, Martinez A, Montero P, Nunez C, Puigdueta I, Quer J, Rivero M, Roset A, Sanchez-Muniz FJ, Vaquero P (2003) Use of calcium or calcium in combination with vitamin D supplementation to prevent fractures and bone loss in people aged 50 years and older: a meta-analysis. Nutricion Hospitalaria 18:109-137
16. Gomes F, Schuetz P, Bounoure L, Austin P, Ballesteros-Pomar M, Cederholm T, Fletcher J, Laviano A, Norman K, Poulia KA, Ravasco P, Schneider SM, Stanga Z, Weekes CE, Bischoff SC (2018) ESPEN guidelines on nutritional support for polymorbid internal medicine patients. Clin Nutr 37 (1):336-353. doi:10.1016/j.clnu.2017.06.025
17. Wolfe RR, Miller SL, Miller KB (2008) Optimal protein intake in the elderly. Clin Nutr 27 (5):675-684. doi:10.1016/j.clnu.2008.06.008
18. Aranceta Bartrina J, Grupo Colaborativo de la Sociedad Espanola de Nutricion C, Arija Val VV, Maiz Aldalur E, Martinez de Victoria Munoz E, Ortega Anta RM, Perez-Rodrigo C, Quiles Izquierdo J, Rodriguez Martin A, Roman Vinas B, Salvador Castell G, Tur Mari JA, Varela Moreira G, Serra Majem L (2016) Dietary Guidelines for the Spanish population (SENC, diciembre 2016); the new graphic icon of healthy food. Nutr Hosp 33 (Suppl 8):1-48. doi:10.20960/nh.827
19. Gray J (2006) Dietary Fibre.
20. EFSA (2010) Scientific Opinion on Dietary Reference Values for water. EFSA Journal 8 (3). doi:10.2903/j.efsa.2010.1459
21. Demontiero O, Herrmann M, Duque G (2011) Supplementation with vitamin D and calcium in long-term care residents. J Am Med Dir Assoc 12 (3):190-194. doi:10.1016/j.jamda.2010.09.013
22. Tang BMP, Eslick GD, Nowson C, Smith C, Bensoussan A (2007) Use of calcium or calcium in combination with vitamin D supplementation to prevent fractures and bone loss in people aged 50 years and older: a meta-analysis. Lancet 370:657-666
23. Dawson-Hughes B, Mithal A, Bonjour JP, Boonen S, Burckhardt P, Fuleihan GE, Josse RG, Lips P, Morales-Torres J, Yoshimura N (2010) IOF position statement: vitamin D recommendations for older adults. Osteoporos Int 21 (7):1151-1154. doi:10.1007/s00198-010-1285-3
24. IOM (1998) Dietary Reference Intakes for Thiamin, Riboflavin, Niacin, Vitamin B6, Folate, Vitamin B12, Pantothenic Acid, Biotin and Choline. National Academy Press,
25. Cichero JA, Lam P, Steele CM, Hanson B, Chen J, Dantas RO, Duivestein J, Kayashita J, Lecko C, Murray J, Pillay M, Riquelme L, Stanschus S (2017) Development of International Terminology and Definitions for Texture-Modified Foods and Thickened Fluids Used in Dysphagia Management: The IDDSI Framework. Dysphagia 32 (2):293-314. doi:10.1007/s00455-016-9758-y
26. Simmons SF, Keeler E, Zhuo X, Hickey KA, Sato HW, Schnelle JF (2008) Prevention of unintentional weight loss in nursing home residents: a controlled trial of feeding assistance. J Am Geriatr Soc 56 (8):1466-1473. doi:10.1111/j.1532-5415.2008.01801.x
27. Forster A, Samaras N, Gold G, Samaras D (2011) Oropharyngeal dysphagia in older adults: A review. European Geriatric Medicine 2 (6):356-362. doi:10.1016/j.eurger.2011.08.007; Wirth R, Dziewas R, Beck AM, Clave P, Hamdy S, Heppner HJ, Langmore S, Leischker AH, Martino R, Pluschinski P, Rosler A, Shaker R, Warnecke T, Sieber CC, Volkert D (2016) Oropharyngeal dysphagia in older persons - from pathophysiology to adequate intervention: a review and summary of an international expert meeting. Clin Interv Aging 11:189-208. doi:10.2147/CIA.S97481; Baijens LW, Clave P, Cras P, Ekberg O, Forster A, Kolb GF, Leners JC, Masiero S, Mateos-Nozal J, Ortega O, Smithard DG, Speyer R, Walshe M (2016) European Society for Swallowing Disorders - European Union Geriatric Medicine Society white paper: oropharyngeal dysphagia as a geriatric syndrome. Clin Interv Aging 11:1403-1428. doi:10.2147/CIA.S107750
28. Cederholm T, Barazzoni R, Austin P, Ballmer P, Biolo G, Bischoff SC, Compher C, Correia I, Higashiguchi T, Holst M, Jensen GL, Malone A, Muscaritoli M, Nyulasi I, Pirlich M, Rothenberg E, Schindler K, Schneider SM, de van der Schueren MA, Sieber C, Valentini L, Yu JC, Van Gossum A, Singer P (2017) ESPEN guidelines on definitions and terminology of clinical nutrition. Clin Nutr 36 (1):49-64. doi:10.1016/j.clnu.2016.09.004; Kondrup J (2003) ESPEN Guidelines for Nutrition Screening 2002. Clinical Nutrition 22 (4):415-421. doi:10.1016/s0261-5614(03)00098-0
29. Simmons SF, Keeler E, An R, Liu X, Shotwell MS, Kuertz B, Silver HJ, Schnelle JF (2015) Cost-Effectiveness of Nutrition Intervention in Long-Term Care. J Am Geriatr Soc 63 (11):2308-2316. doi:10.1111/jgs.13709
30. Simmons SF, Hollingsworth EK, Long EA, Liu X, Shotwell MS, Keeler E, An R, Silver HJ (2017) Training Nonnursing Staff to Assist with Nutritional Care Delivery in Nursing Homes: A Cost-Effectiveness Analysis. J Am Geriatr Soc 65 (2):313-322. doi:10.1111/jgs.14488
